# A Quality Improvement Initiative for Inpatient Advance Care Planning

**DOI:** 10.1001/jamahealthforum.2024.3172

**Published:** 2024-10-04

**Authors:** Olivia A. Sacks, Megan Murphy, James O’Malley, Nancy Birkmeyer, Amber E. Barnato

**Affiliations:** 1 Department of Surgery, Boston Medical Center, Boston, Massachusetts; 2 Boston University Chobanian & Avedisian School of Medicine, Boston, Massachusetts; 3 The Dartmouth Institute for Health Policy and Clinical Practice, Geisel School of Medicine at Dartmouth, Lebanon, New Hampshire; 4 Department of Biomedical Data Sciences, Geisel School of Medicine at Dartmouth, Lebanon, New Hampshire; 5 Section of Palliative Care, Department of Medicine, Geisel School of Medicine at Dartmouth and Dartmouth Health, Lebanon, New Hampshire

## Abstract

**Importance:**

The Centers for Medicare & Medicaid Services (CMS) implemented advance care planning (ACP) billing codes in 2016 to encourage practitioners to conduct and document ACP conversations, and included ACP as a quality metric in the CMS Bundled Payments for Care Improvement Initiative in 2018. Use of this billing code in the inpatient setting has not been studied.

**Objective:**

To determine whether a quality improvement intervention to increase inpatient ACP is effective in increasing ACP billing rates or changing hospital treatment plans or patient outcomes.

**Design, Settings, and Participants:**

This nationwide cohort study and difference-in-differences analyses compared changes in ACP billing, treatment, and outcomes in Medicare fee-for-service beneficiaries aged 65 years and older who were hospitalized and cared for by 3 different groups: practitioners employed by a national acute care staffing organization who underwent an ACP quality improvement intervention, nonintervention practitioners at the same hospital, and control group practitioners from other hospitals. Using data from the Master Beneficiary Summary File, acute care hospital stays for nonsurgical conditions were linked to Medicare enrollment, claims, and vital status data from 1-year preadmission to 1-year postadmission from 2015 to 2019. The ACP billing rates for each group were assessed for associations with 6 inpatient treatments and 4 outcomes. Data analyses were performed from January 2022 to December 2024.

**Main Outcomes and Measures:**

Billed ACP conversations, receipt of intensive care and life support (intensive care unit admission, gastrostomy tube placement, mechanical ventilation, tracheostomy), treatment limitations (newly initiated do-not-resuscitate orders) and outcomes (discharge to hospice, inpatient death, 30-day postadmission death, and 1-year postadmission death).

**Results:**

The total study sample included 109 intervention hospitals, 1691 control hospitals, nearly 12 million Medicare fee-for-service beneficiaries aged 65 years and older, and 738 309 practitioners associated with admissions from 2016 to 2018. ACP billing rates increased more for the intervention (1.3% in preintervention to 14.0% in postintervention; *P* < .001) than for the nonintervention (same hospitals) and control groups (odds ratio [OR], 2.6; 95% CI, 1.7-4.1 intervention vs control). Increased ACP billing rates were significantly associated with decreased rates of inpatient death in the intervention group (OR, 0.95; 95% CI, 0.90-1.00) compared to the nonintervention (OR, 1.10; 95% CI, 1.04-1.17) and control groups (reference). All other associations were nonsignificant.

**Conclusions and Relevance:**

This nationwide cohort study suggests that while the ACP quality initiative increased ACP billing, changes in clinical outcomes were inconsistent with the hypotheses. Further study is needed to address questions regarding confounding by unobserved measures of care quality.

## Key Points

**Question:**

Did a US nationwide quality improvement initiative led by an acute care staffing organization increase inpatient advance care planning (ACP) rates or affect hospital treatment plans and patient outcomes?

**Findings:**

This cohort study including 109 intervention hospitals, 1691 control hospitals, nearly 12 million Medicare beneficiaries aged 65 years and older, and 738 309 practitioners with hospital admissions from 2016 to 2018 found that a quality improvement initiative to increase inpatient ACP was associated with increased ACP billing rates, but not with changes in treatment plans or improved patient outcomes.

**Meaning:**

These findings suggest that increasing the uptake of ACP in the inpatient setting may not alter hospital treatment plans or patient outcomes.

## Teaser

This nationwide cohort study evaluates the effectiveness of a quality improvement intervention to improve inpatient advance care planning by assessing billing rates and changes in treatment plans and patient outcomes.

## Introduction

Advance care planning (ACP) provides an opportunity for clinicians to elicit the goals and values of patients regarding current and future medical care. ACP improves alignment between patient preferences and their medical treatment.^1,2^ Ideally, ACP occurs iteratively in an outpatient setting and includes the completion of advance directives, but only 1 in 3 US adults have any documentation describing their end-of-life wishes.^3,4,5^

Because most patients do not have established advance care plans, conversations about treatment preferences often happen in the acute care setting. Hospital-based practitioners meet patients during key moments in the care trajectory that may reflect a major inflection in health status. Without established relationships with the stakeholders, these practitioners are tasked with implementing time-sensitive treatment while also ascertaining which treatments align with the patient’s goals. However, the frequency with which ACP occurs in the acute care setting varies widely, and the prevalence is low overall.^6,7,8,9,10^

In 2016, the Centers for Medicare & Medicaid Services (CMS) implemented ACP billing codes to incentivize all practitioners to conduct ACP. Early studies have shown that the incidence of billed ACP is low in the acute care setting and that the billing codes alone have not substantially affected the uptake of ACP billing or practice.^10,11^ Some clinician groups, particularly those involved with the CMS Bundled Payments for Care Improvement Initiative−advanced (BPCI-A), which includes ACP as a quality metric alongside risk-adjusted mortality, have made a more targeted effort to increase ACP with focused education and awareness initiatives. To our knowledge, how these efforts have affected ACP rates has not been studied. This study sought to understand how a specific ACP quality improvement (QI) intervention affected ACP billing rates in a large national acute care practitioner staffing organization (PSO). We hypothesized that the ACP QI intervention would increase ACP billing; that there would be spillover impacts from practitioners employed by the PSO compared with those not employed by the PSO practicing in the same hospital; and that greater ACP rates would be associated with lower use of invasive mechanical ventilation, an increase in do-not-resuscitate (DNR) orders that would prompt an increase in inpatient death rates and in hospice discharges.

## Methods

This study was approved by the Dartmouth College Committee for the Protection of Human Subjects. Informed consent was waived because this was a large and retrospective study using only deidentified data. The manuscript was prepared according to the Strengthening the Reporting of Observational Studies in Epidemiology (STROBE) reporting guideline.

### Data Sources

We used Medicare fee-for-service enrollment, Parts A and B (Master Beneficiary Summary File [MBSF]), and claims data for patient-level demographic and enrollment characteristics, diagnoses, and service use, including ACP. We used Part A claims for hospital billing information and Part B claims for practitioner billing information. We identified admissions for acute care hospital stays for nonsurgical conditions between January 1, 2016, and December 31, 2018, using MedPAR records. We linked admission data to enrollment, claims, and death data to create longitudinal beneficiary histories from 1 year before the index hospitalization to 1-year postadmission. We used personnel records from the acute care PSO to identify employed physicians and advanced practice practitioners (APPs) and contracted hospitals. We used the American Hospital Association Annual Survey data for the most current year available for each hospital during the study period to identify select hospital characteristics. We used data from the PSO about hospital BPCI participation in conjunction with hospital information found on inpatient claims to identify hospitals enrolled in BPCI and BPCI-A. We used the National Plan & Provider Enumeration System look-up tool to identify practitioners.

### Study Time Periods

Three study periods were defined. The first was the preintervention period (January 1-June 30, 2016), after CMS introduced ACP billing codes but before the start of the ACP QI initiative. The second was the intervention period (July 1, 2016, to June 30, 2017) when the QI intervention was rolled out to hospitalists at hospitals staffed by the PSO. The third was the postimplementation period (July 1, 2017, to December 30, 2018) after the QI initiative had been fully implemented.

### Description of the PSO and the QI Intervention

The PSO is a physician-led for-profit staffing organization that contracts directly with hospitals to provide acute care physicians and APPs (emergency medicine, hospitalist, and intensive care unit [ICU] staff), competing on contract cost and quality of patient outcomes. At the time of this study, the PSO had more than 2500 employees in more than 250 hospitals in 41 states. Approximately half of the hospitals staffed by the PSO were enrolled in BPCI and/or BPCI-A, and the PSO hospital contract included gainsharing.

Shortly after the introduction of ACP billing codes in 2016, the PSO initiated an ACP QI effort to increase use of ACP and ACP billing, especially for patients 65 years and older, those at high risk for dying in the next year, and/or those having experienced functional status changes that affect quality of life and independence. The ACP QI program included mandatory education in the use of ACP billing codes; small financial incentives for ACP documentation ($20); requiring practitioners to answer the surprise question (“Would I be surprised if this patient died in the next 12 months?”) for patients at admission—to prime them to reflect on the patient’s risk of dying during the next year; a resource nurse dedicated to encouraging hospitalists to complete an ACP with any patient who was “positive” on the surprise question screening; and monthly audit and feedback of ACP billing rates to the individual hospitalists and their hospital management. These interventions were restricted to the subset of practitioners at the hospital employed by the PSO.

### Study Sample

The sample comprised intervention and control hospitals and intervention, nonintervention, and control practitioners. The Figure summarizes hospital, practitioner, and stay sampling.

**Figure.**
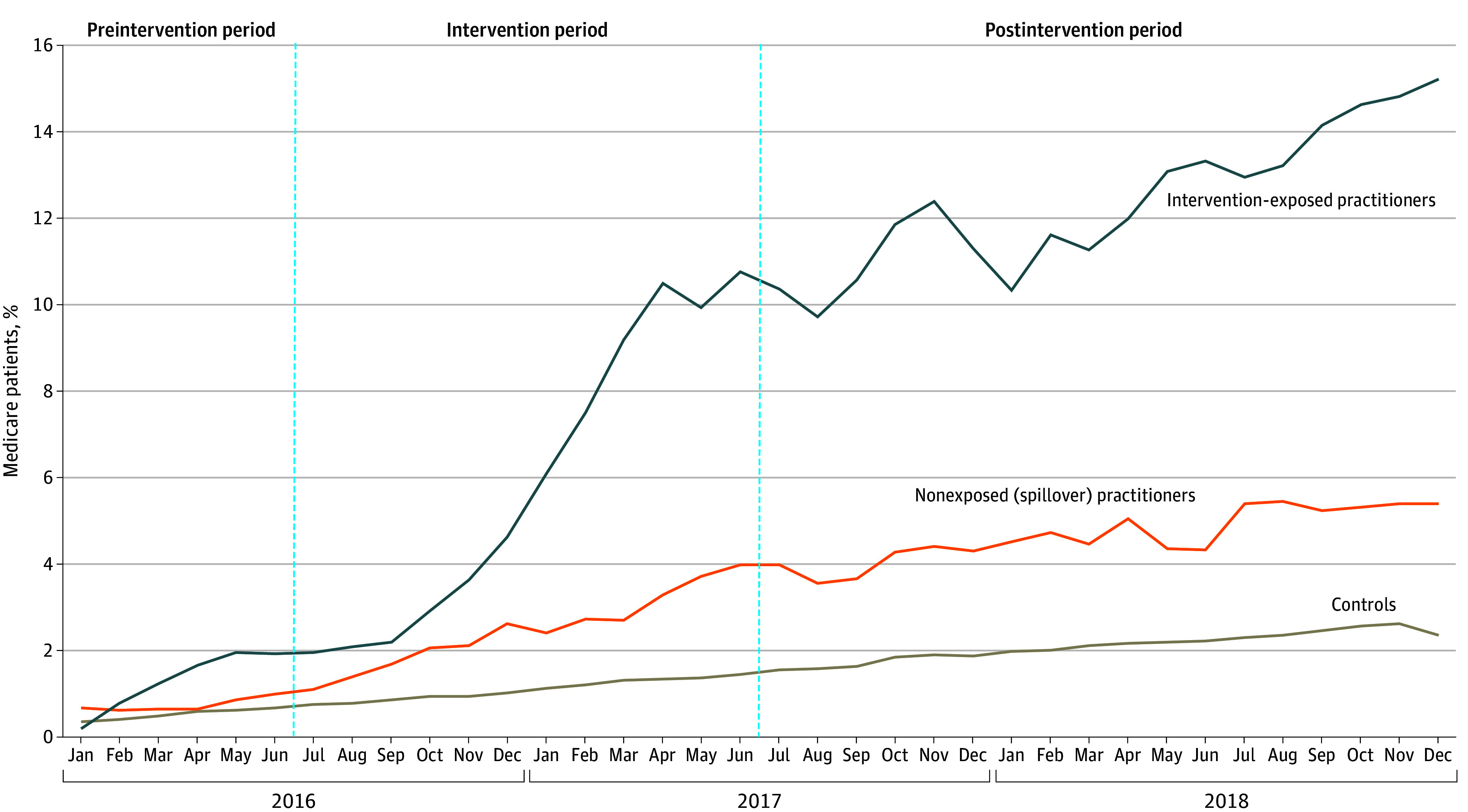
Trends in Billing for Advance Care Planning (ACP) Conversations With Medicare Beneficiaries During Inpatient Stays in 2016 to 2018, by Hospital Type ^a^Spillover was intended to capture professional influence; that is, when intervention practitioners (exposed to the initiative to increase ACP conversations and billing) discussed the effort with (ie, exposing) nonintervention practitioners, influencing them to begin billing for ACP discussions. The blue lines demarcate the time periods.

#### Intervention and Control Hospitals

We defined intervention hospitals as those participating in BPCI and staffed by the PSO from January 1, 2016, to December 31, 2018. Although ACP was not incentivized in the BPCI until the introduction of BPCI-A in 2018, value-based payment programs may affect changes in health care utilization. Then, we defined control hospitals as all the remaining hospitals participating in BPCI that were not staffed by the PSO during the same time period. We had no information on hospitalist staffing models or QI initiatives at control hospitals.

#### Intervention, Nonintervention, and Control Practitioners

We identified physicians and APPs (hereafter, practitioners) at intervention and control hospitals using billing National Provider Identifiers (NPI) linked through the National Plan & Provider Enumeration System look-up tool.^12^ At the intervention hospitals, we distinguished practitioners employed by the PSO—who would have undergone the ACP QI initiative—from those who were not, using PSO staffing records. We hypothesized that the practitioners not employed by the PSO who interact with practitioners participating in the QI intervention could be subject to spillover effects.^13^ The concept of spillover in this study was described in terms of professional influence. For example, if intervention practitioners employed by the PSO discussed ACP billing with nonintervention practitioners at the same hospital, that could influence nonintervention practitioners to begin billing for ACP. We have no information about these nonintervention practitioners. We assume them to be community practitioners with hospital admitting privileges who do not cede their patients’ care to the hospitalists contracted by the hospital. Similarly, we have no information regarding the control practitioners at control hospitals, including whether they are part of a hospital-employed hospitalist group, employed by a competing acute care PSO hospitalist group, or are community-based practitioners.

### Patients Admitted to Intervention and Control Hospitals

We identified hospital stays at intervention and control hospitals through MedPAR records restricted to inpatient claims (ie, excluding observation stays). We identified practitioner Part B encounter claims associated with these records using admission and discharge dates. Beneficiaries admitted to selected hospitals were included in the study population if they were aged 65 years or older at the time of their admission and continuously enrolled in Medicare Parts A and B for at least 12 months before hospitalization.

### Treatment Groups

We classified inpatient stays at intervention and control hospitals into 3 treatment groups based on the treating practitioner: intervention practitioners, nonintervention practitioners at hospitals staffed by the PSO (ie, those subject to possible spillover), and control practitioners. We classified stays as an intervention stay if any of the Part B encounter was billed to an NPI linked to the PSO. Nonintervention stays were defined as those occurring at an intervention hospital but without evidence of billing by the practitioners employed by the PSO. Control stays were defined as those occurring at control hospitals.

### Primary and Secondary Outcomes

The primary outcome for this study was billed ACP, indicated by the Healthcare Common Procedure Coding System billing code 99497 in the Part B medical claim. Additional details are available in eAppendix 1 in Supplement 1.

Secondary outcomes were chosen by clinicians and included variables related to life-sustaining treatment, treatment limitation, and survival: ICU admission, gastrostomy tube placement for enteral feeding, invasive and noninvasive mechanical ventilation, tracheostomy, newly initiated DNR orders, discharge to hospice, inpatient death, 30-day postadmission death, and 1-year postadmission death. eAppendix 2 in Supplement 1 provides the relevant procedure codes.

### Covariates

Covariates describing general patient characteristics on admission included age, sex, and dual Medicare-Medicaid enrollment status. Age at admission was calculated as the difference in the admission date and the beneficiary’s date of birth and was classified into 3 categories (age 65-74, 75-84, and ≥85 years). Sex was ascertained from the MBSF file. Dual eligibility status was assessed at the time of admission using the dual enrollment indicator (codes 02, 03, 04, 05, 06, 08, 09) associated with the month of admission from the MBSF file. To capture clinical comorbidities, we used a 1-year lookback to calculate a predicted mortality risk at admission score, using a modified approach to a validated model developed by van Walraven^14,15^ (eAppendix 3 in Supplement 1). To capture hospital practice patterns, we calculated each hospital’s ACP billing rate as a time-varying variable by computing the proportion of patients that had at least 1 ACP conversation during their hospital stay.

### Statistical Analysis

We used a difference-in-difference (DID) approach to evaluate the association of the QI intervention with ACP billing rates between the intervention and control groups with the goal of minimizing biases from time-invariant differences between the populations and to control for shared secular trends across the entire population. We performed a test of parallel trends in the preperiod, and ACP rates were parallel between the intervention and control groups, supporting the appropriateness of the use of the DID approach (Figure).

The dependent variables in these models were constructed as dichotomous variables. Therefore, to adjust for clustering by hospital, we estimated the effect of the treatment by an intervention practitioner on ACP billing rates using the generalized estimating equations procedure assuming a negative binomial outcome distribution with a Bernoulli distribution with a logit link (for binary outcomes). The independent variables were patient age, sex, dual Medicare-Medicaid eligibility, predicted mortality risk score, admission month, exposure group, intervention period, and the interaction of each treatment group and study period. The inclusion of the patient demographic and mortality risk variables sought to account for confounding by observed factors.

The difference in the changes over time among the groups netted out the amount of change over time from the pre- to the given time period (either intervention or postintervention) that can be attributed to the intervention’s impact on the treatment group compared with the control. With 3 treatment groups and 3 time periods, interactions comprised 4 degrees of freedom; we reported the omnibus test of any interaction as well as the treatment group by time period (vs preintervention as baseline) with individual DID-effect regression coefficients and their 95% CIs and *P* values. In a separate series of models with treatment indicators and outcome measures as dependent variables, we examined the extent to which the ACP billing rate for each treatment group accounted for any association between the QI initiative and these dependent variables, providing insights into the extent to which ACP billing mediated the impact of the intervention.

In all of the analyses, statistical significance was determined from the confidence intervals for the difference-in-difference term’s coefficient (ie, 95% CIs that did not overlap with 0 indicated statistical significance at the 5%). Each confidence interval presents the range of effect sizes that are supported by the data, irrespective of whether the estimated effect was found to be statistically significantly different from 0, and so are commensurate with a goal of reporting results in a descriptive or exploratory tone as opposed to a confirmatory testing-based manner. However, a Bonferroni test corrected levels provided a landmark against which *P* values were compared to indicate which ones (if any) withstood a conservative adjustment for multiple comparisons. With 10 secondary outcomes, a threshold of *P* = .05 for an individual statistical test was reduced to approximately .005 under the Bonferroni adjustment. Statistical tests were 2-tailed and analyses were performed using SAS, version 14.0 (SAS Institute Inc). Data analyses were performed from January 2022 to December 2024.

## Results

### Sample Characteristics

The total study sample included 109 intervention hospitals, 1691 control hospitals, nearly 12 million Medicare fee-for-service beneficiaries aged 65 years and older, and 738 309 unique performing NPIs associated with admissions from 2016 to 2018 (10.2% of the practitioners appeared in more than 1 cohort during the 3-year period). The intervention and control hospitals had similar characteristics (eAppendix 1 in Supplement 1). Due to the large sample size, nearly all the differences in characteristics of patients admitted to intervention compared to control hospitals were statistically significant (Table 1). However, with few exceptions, these differences were small and not clinically significant (absolute difference, <2 percentage points). For example, patients at intervention-practitioner−staffed hospitals were less likely to be dually eligible for Medicare and Medicaid (19.4% for the intervention group and 18.3% for nonintervention [spillover] group) compared to those in the control group (20.4%).

**Table 1.** Patient Characteristics, by Hospital and Treating Practitioner Type, 2016 to 2018

Characteristic	Treatment type, No. (%)			*P *value
	Control hospital and practitioner	Intervention hospital		
		Nonintervention (spillover^a^) practitioner	Intervention practitioner	
Total patients, No.	11 477 716	285 771	431 819	NA
Unique Medicare beneficiaries, No.	6 052 896	188 375	279 002	NA
Eligible hospital stays				
1	3 527 942 (58.3)	135 862 (72.1)	194 509 (69.7)	<.001
2	1 284 905 (21.2)	30 966 (16.4)	50 317 (18.0)	
3	578 062 (9.6)	11 269 (6.0)	18 407 (6.6)	
≥4	661 987 (10.9)	10 278 (5.5)	15 769 (5.7)	
**Demographic characteristics at admission**				
Age, y				
65-74	4 077 017 (35.5)	105 362 (36.9)	154 201 (35.7)	<.001
75-84	4 149 943 (36.2)	103 374 (36.2)	158 048 (36.6)	
≥85	3 250 756 (28.3)	77 035 (27.0)	119 570 (27.7)	
Sex				
Female	6 445 449 (56.2)	160 274 (56.1)	242 460 (56.1)	
Male	5 032 267 (43.8)	125 497 (43.9)	189 359 (43.9)	.75
Dual eligible				
No	9 032 710 (78.7)	230 599 (80.7)	343 751 (79.6)	<.001
Yes	2 338 960 (20.4)	52 322 (18.3)	83 772 (19.4)	
Unknown	106 046 (0.9)	2850 (1.0)	4296 (1.0)	
**Health status at admission, per CCI score**				
MI	2 246 780 (19.6)	56 723 (19.8)	83 872 (19.4)	<.001
CHF	5 018 831 (43.7)	127 226 (44.5)	186 009 (43.1)	<.001
PVD	4 853 514 (42.3)	121 002 (42.3)	172 768 (40.0)	<.001
CVD	3 884 572 (33.8)	98 214 (34.4)	139 261 (32.2)	<.001
Dementia	2 603 275 (22.7)	61 888 (21.7)	93 622 (21.7)	<.001
CRD	5 312 062 (46.3)	131 987 (46.2)	200 530 (46.4)	.07
Mild liver disease	1 285 647 (11.2)	32 062 (11.2)	45 300 (10.5)	<.001
Diabetes (no complications)	4 932 557 (43.0)	127 098 (44.5)	183 762 (42.6)	<.001
Diabetes (complications)	3 250 144 (28.3)	84 659 (29.6)	124 275 (28.8)	<.001
Hemi/paraplegia	675 856 (5.9)	18 565 (6.5)	25 587 (5.9)	<.001
Kidney disease	4 560 648 (39.7)	116 997 (40.9)	169 664 (39.3)	<.001
Cancer (NM)	2 712 731 (23.6)	64 716 (22.6)	91 476 (21.2)	<.001
Moderate/severe LD	247 298 (2.2)	6822 (2.4)	8872 (2.1)	<.001
Cancer (M)	801 885 (7.0)	18 625 (6.5)	26 346 (6.1)	<.001
HIV/AIDS	29 732 (0.3)	661 (0.2)	768 (0.2)	<.001
PMRS, mean (SD)	0.33 (0.20)	0.33 (0.20)	0.33 (0.19)	<.001

Abbreviations: CCI, Charlson Comorbidity Index; CHF, congestive heart failure; CRD, chronic respiratory disease; CVD, cerebrovascular disease; LD, liver disease; M, metastatic; MI, myocardial infarction; NA, not applicable; NM, nonmetastatic; PMRS, predicted mortality risk score; PVD, peripheral vascular disease.

^a^ Spillover was intended to capture professional influence; that is, when intervention practitioners (exposed to the initiative to increase ACP conversations and billing) discussed the effort with (ie, exposing) nonintervention practitioners, influencing them to begin billing for ACP discussions.

### ACP Rates

For patients treated in the intervention group, unadjusted ACP billing rates increased from 1.3% in the preintervention period to 14.0% in the postintervention period (*P* < .001). For patients in the nonintervention group at the same hospitals (practitioners potentially subject to spillover), unadjusted ACP billing rates increased from 0.7% to 4.9% (*P* < .001). For patients in the control group, unadjusted ACP billing rates increased from 0.5% to 2.1% (*P* < .001) (Figure). In risk-adjusted analyses, increases in ACP billing across time (Table 2) were significantly greater for the intervention group (OR, 2.6; 95% CI, 1.7-4.1; *P* < .001) and the nonintervention group (odds ratio [OR], 1.6; 95% CI, 1.0-2.6; *P* = .06) than in the control group, confirming a spillover association of the intervention from practitioner-to-practitioner within hospitals staffed by the PSO.

**Table 2.** Advanced Care Planning (ACP) Billing Rates, Inpatient Treatments, and Patient Outcomes, by Hospital and Treating Practitioner Type, 2016 to 2018

Treatment or outcome	Treatment type, No. (%)								
	Control hospital and practitioner (n = 11 477 716)			Intervention hospital					
				Nonintervention (spillover^a^) practitioner (n = 285 771)			Intervention practitioner (n = 431 819)		
	Preintervention (n = 1 940 380)	Intervention (n = 3 872 727)	Postintervention (n = 5 664 609)	Preintervention (n = 51 062)	Intervention (n = 96 778)	Postintervention n = 137 931	Preintervention (n = 69 061)	Intervention (n = 145 867)	Postintervention (n = 216 891)
**Primary outcome**									
ACP billing	9205 (0.5)	40 739 (1.1)	116 405 (2.1)	352 (0.7)	2353 (2.4)	6384 (4.6)	860 (1.2)	8871 (6.1)	26 629 (12.3)
**Secondary outcomes**									
Tracheostomy	1073 (0.1)	2086 (0.1)	3269 (0.1)	16 (0)	38 (0)	52 (0)	<11 (0)	31 (0)	37 (0)
ICU	662 332 (34.1)	662 332 (34.1)	1 910 715 (33.7)	20 107 (39.4)	37 268 (38.5)	51 647 (37.4)	27 297 (39.5)	57 220 (39.2)	86 572 (39.9)
Gastrostomy	20 434 (1.1)	40 124 (1.0)	54 134 (1.0)	587 (1.1)	1023 (1.1)	1365 (1.0)	670 (1.0)	1465 (1.0)	1962 (0.9)
Invasive MV	65 909 (3.4)	132 732 (3.4)	187 900 (3.3)	2037 (4.0)	4009 (4.1)	5719 (4.1)	2483 (3.6)	5107 (3.5)	7246 (3.3)
Noninvasive MV	61 430 (3.2)	127 754 (3.3)	204 701 (3.6)	1622 (3.2)	3367 (3.5)	5774 (4.2)	2793 (4.0)	5928 (4.1)	8949 (4.1)
New DNR order	50 042 (2.6)	115 026 (3.0)	194 649 (3.4)	1406 (2.8)	2955 (3.1)	5229 (3.8)	1826 (2.6)	4300 (2.9)	6717 (3.1)
Discharge to hospice	88 165 (4.5)	181 970 (4.7)	272 384 (4.8)	2479 (4.9)	4649 (4.8)	6660 (4.8)	3289 (4.8)	7165 (4.9)	10 780 (5.0)
Inpatient death	81 148 (4.2)	160 779 (4.2)	229 084 (4.0)	2362 (4.6)	4671 (4.8)	6671 (4.8)	2944 (4.3)	5961 (4.1)	8551 (3.9)
Death within 1 mo	216 340 (11.1)	434 181 (11.2)	624 107 (11.0)	6038 (11.8)	11 522 (11.9)	16 450 (11.9)	8028 (11.6)	17 205 (11.8)	24 632 (11.4)
Death within 1 y	646 664 (33.3)	1 303 189 (33.7)	1 885 350 (33.3)	17 293 (33.9)	32 880 (34.0)	46 435 (33.7)	23 168 (33.5)	49 624 (34.0)	73 232 (33.8)

Abbreviations: DNR, do-not-resuscitate; MV, mechanical ventilation; ICU, intensive care unit.

^a^ Spillover was intended to capture professional influence; that is, when intervention practitioners (exposed to the initiative to increase ACP conversations and billing) discussed the effort with (ie, exposing) nonintervention practitioners, influencing them to begin billing for ACP discussions.

### Secondary Outcomes

We summarize rates of ACP, treatment, and outcomes in the pre- and postintervention periods for the control group, nonintervention group, and intervention group in Table 2. After accounting for multiple comparisons, the only statistically significant association between increases in billed ACP and outcomes, by treatment group, was inpatient death. As shown in Table 3, compared with the control group, increasing ACP rates was associated with decreased inpatient death in the intervention group (OR, 0.95; 95% CI, 0.90-1.00; *P* = .04) but increased inpatient death in the nonintervention group (OR, 1.10; 95% CI, 1.04-1.17; *P* = .001) (*P* for 3-group comparison = .003). The inclusion of the hospital-by-treatment group ACP billing rate as a predictor accounted for little to none of the marginal association between exposure to the ACP QI initiative and inpatient death (ie, there was a small decrease in the OR for inpatient death, from 0.95 to 0.93). This suggests that the mechanism by which treatment and outcomes were impacted was largely not through changes in ACP billing. There were no statistically significant differences in rates of other secondary outcomes associated with increased ACP billing among the patients treated by intervention practitioners compared with controls. These included tracheostomy (OR, 1.4; 95% CI, 0.58-3.4; *P* = .45), ICU admission (OR, 1.03; 95% CI, 0.95-1.12; *P* = .46), gastroenterostomy tube placement (OR, 1.03; 95% CI, 0.92-1.14; *P* = .64), noninvasive mechanical ventilation (OR, 0.89; 95% CI, 0.79-0.96; *P* = .03), invasive mechanical ventilation (OR, 0.94; 95% CI, 0.88-1.01; *P* = .08), discharge to hospice (OR, 0.98; 95% CI, 0.91-1.06; *P* = .64), 1-month mortality (OR, 0.98; 95% CI, 0.95-1.01; *P* = .17), on 1-year mortality (OR, 1.01; 95% CI, 0.98-1.04; *P* = .53), or new DNR order (OR, 0.87; 95% CI, 0.79-0.96; *P* = .008; overall *P* = .03).

**Table 3.** Risk-Adjusted Odds of Advanced Care Planning (ACP) Billing Rates, Treatments, and Postdischarge Patient Outcomes, by Intervention and Nonintervention Practitioners at the Same Intervention Hospital Compared With Control Practitioners and Hospitals

Treatment or outcome	Group					Overall *P* value
	Control	Nonintervention (spillover^a^)		Intervention		
		OR (95% CI)	*P* value	OR (95% CI)	*P* value	
**Primary outcome**						
ACP billing	1 [Reference]	1.60 (0.99-2.60)	.06	2.61 (1.67-4.07)	<.001	<.001
**Secondary outcomes**						
Tracheostomy	1 [Reference]	1.16 (0.66-2.03)	.61	1.40 (0.58-3.38)	.45	.68
ICU	1 [Reference]	0.94 (0.82-1.08)	.38	1.03 (0.95-1.12)	.46	.34
Gastrostomy	1 [Reference]	0.95 (0.85-1.07)	.42	1.03 (0.92-1.14)	.64	.54
Invasive MV	1 [Reference]	1.08 (1.00-1.16)	.06	0.94 (0.88-1.01)	.08	.05
Noninvasive MV	1 [Reference]	1.17 (0.85-1.61)	.33	0.89 (0.79-0.99)	.04	.03
New DNR order	1 [Reference]	1.04 (0.82-1.33)	.72	0.87 (0.79-0.96)	.008	.03
Discharge to hospice	1 [Reference]	0.95 (0.88-1.01)	.11	0.98 (0.91-1.06)	.64	.30
Inpatient death	1 [Reference]	1.10 (1.04-1.17)	.001	0.95 (0.90-1.00)	.04	.003
Death within 1 mo	1 [Reference]	1.04 (0.99-1.09)	.08	0.98 (0.95-1.01)	.17	.09
Death within 1 y	1 [Reference]	1.00 (0.97-1.04)	.81	1.01 (0.98-1.04)	.53	.82

Abbreviations: DNR, do-not-resuscitate; ICU, intensive care unit; MV, mechanical ventilation; OR, odds ratio.

^a^ Spillover was intended to capture professional influence; that is, when intervention practitioners (exposed to the initiative to increase ACP conversations and billing) discussed the effort with (ie, exposing) nonintervention practitioners, influencing them to begin billing for ACP discussions.

## Discussion

In a national study of nearly 12 million patients treated by approximately 800 000 practitioners at 1800 hospitals in the US, we found that a physician-directed ACP QI initiative implemented by a national acute care PSO was associated with an increased likelihood of an ACP conversation being billed (all else being equal) for Medicare beneficiaries. To our knowledge, these are the first data indicating improvements in billed ACP associated with a comprehensive physician-directed ACP QI initiative. Given generally conservative preferences for life-sustaining treatment, we had hypothesized that increases in ACP billing—to the extent they represent increases in the occurrence of underlying ACP conversations—would be associated with a decrease in receipt of intensive care and life support (ICU admission, gastrostomy tube placement, noninvasive and invasive mechanical ventilation, tracheostomy); an increase in treatment limitations (newly initiated DNR orders); and an associated increase in discharge to hospice, inpatient death, 30-day postadmission death, and 1-year postadmission death. Our analysis demonstrated that an increase in the ACP billing rate was not statistically significantly associated with these outcomes (with the exception of inpatient death) and may be attributable to other characteristics of the intervention hospitals and practitioners (or patients).

Rates of ACP billing since the introduction of the billing codes in 2016 have been low overall. A 2022 study on Medicare beneficiaries^16^ from more than 50 000 outpatient medical practices from 2016 to 2018 found that only 15% of these practices billed for ACP. Although this study is not comparable to ours because it used physician practices as the unit of analysis and it was conducted in the outpatient setting, it does serve to underscore the low uptake of ACP billing codes overall. We similarly find sluggish uptake of ACP billing codes in control hospitals where less than 2% of hospitalized Medicare beneficiaries aged 65 years and older had an ACP encounter billed in those hospitals in 2018. This is compared to intervention practitioners who increased billing code use to nearly 16% of their admissions by the end of 2018 (Figure).

This study adds to the existing literature on ACP in 3 ways. To our knowledge, it is the first study to demonstrate the impact of a comprehensive ACP QI intervention on ACP billing rates. Second, it reports on a much larger scale ACP QI intervention than previously conducted or reported in the literature—affecting more than 1 million hospitalized Medicare beneficiaries. Third, this QI intervention focused on the inpatient setting in contrast to most ACP interventions, which have focused on the outpatient setting.

Surprisingly, significant increases in ACP billing did not reduce inpatient treatment intensity (ie, invasive mechanical ventilation, tracheostomy, gastrostomy) or increase DNR orders and discharges to hospice. Our study stands in contrast to 2 recent studies. Weissman et al^17^ examined the association of ACP visits with 5 measures of end-of-life intensity, limited only to decedents. Billed ACP was uncommon, but the patients who did receive billed ACP were found to have lower overall end-of-life intensity. In the other study, Gupta et al^18^ performed a similar analysis that assessed Medicare fee-for-service decedents in 2017 for evidence of ACP billing. The small number of decedents with a billed ACP encounter experienced less intensive end-of-life care, defined as reduced odds of hospitalizations, emergency department visits, and ICU stays within 1 month of death, and lower likelihood of dying in the hospital. Perhaps, because Gupta et al selected decedents only, patients closer to death may experience more meaningful changes in clinical care trajectory from ACP conversations. Also, this study’s^18^ measure of end-of-life care may have been more rigorous than the Medicare measures we used. For example, the DNR diagnosis code in the Medicare data may not be used uniformly for DNR orders, and so may not accurately reflect the code status of the patients in our study.

There are a few possible explanations for our finding that there was no statistically significant association between increased ACP billing and most of the secondary outcomes. First, perhaps the practitioners exposed to the QI intervention were already performing ACP conversations before the QI intervention. Thus, the increase in ACP billing did not change practice, but rather changed documentation and billing for work they were already completing. Two recent qualitative studies^19,20^ assessed practitioners’ attitudes toward ACP billing and found that a major barrier was the act of billing itself, adding extra work and time that was difficult to integrate into inpatient workflow. It is possible that the practitioners in our study were similarly performing ACP the entire time, but the QI intervention, organizational encouragement, and individual incentives encouraged them to bill for a service they were already providing.

Second, it is possible that billed ACP conversations in the inpatient setting do not change health care delivery. There has been considerable debate recently in the literature^21^ regarding the effectiveness of ACP for changing health care delivery outcomes. Much of the literature references ACP conversations in the outpatient setting—casting doubt on whether planning for hypothetical health-related decisions in advance changes real-time decision-making. In contrast, we had assumed that inpatient ACP was likely to reflect goals of care conversations, which affect near-term and nonhypothetical decisions, and therefore, are more likely to be associated with changes in health care delivery.

The heterogeneity of the association of increased ACP billing with inpatient death between the intervention (reduced odds of death) and the nonintervention groups (increased odds of death) is systematically different in the 2 groups. This could be a marker of QI-adherent PSO-employed practitioners in the treatment group, a proxy for conscientiousness that may be correlated with clinical quality, or it could be a marker of patient illness severity in the nontreatment group.

### Strengths and Limitations

This study has many strengths. We used a robust measure of ACP: a 16-minute or longer conversation that was documented and billed with an ACP code. The ACP QI initiative used multiple extrinsic QI strategies known to modify practitioner behavior, including education, financial incentives, priming, multidisciplinary support, and audit and feedback.^22^ It targeted the well-studied barriers to ACP, such as discomfort with the topic, lack of training on the subject, and the absence of reimbursement.^23,24,25,26^ Our DID approach included 2 types of comparators—ie, practitioners at the same hospital and practitioners at other similar hospitals—and rigorous controls for illness severity, including a 1-year lookback risk of mortality measure (eAppendix 4 in Supplement 1).

Despite these exacting methods, our study has many limitations. First and most importantly, we are unable to conclude whether the increase in ACP reflects a true increase in the frequency of these conversations by intervention practitioners, or simply reflects changes in their billing behavior (low sensitivity). However, billed conversations tend to be a robust measure of ACP because the clinicians have met the time requirements and decided to spend extra time performing billing (high specificity). Therefore, our study likely underestimates the true number of ACP conversations occurring in the hospital. Additionally, while we hypothesized that the QI initiative explains the significant increase in ACP rates in our intervention population, causal inference is limited because of the observational nature of the study. Lastly, we were not able to obtain demographic, education, or employment information on the practitioners included in the study, data that may have helped to delineate some of the observed differences in clinical practice.

## Conclusions

A comprehensive physician-directed ACP QI initiative was associated with increased incidence of ACP billing in the acute care setting, but these changes were not statistically significantly associated with inpatient treatment intensity or outcomes, with the exception of a heterogeneous effect on inpatient mortality. These unexpected findings raise questions about using ACP billing as a proxy for ACP conversations and/or whether billed ACP encounters in the inpatient setting meaningfully affect clinical treatment or outcomes for older adults who are severely ill and hospitalized.

## Notes

Supplement 1. eAppendix 1. Characteristics of intervention and control hospitals eAppendix 2. Billing Code 99497 eAppendix 3. Tracheostomy Procedure Codes eAppendix 4. Mortality Risk Score

Supplement 2. Data Sharing Statement
