# Supplementary material for: A Quality Improvement Initiative for Inpatient Advance Care Planning
Source: JAMA Health Forum. 2024 Oct 4;5(10):e243172. doi: 10.1001/jamahealthforum.2024.3172 (PMC11452818; doi:10.1001/jamahealthforum.2024.3172)
Supplement: Supplement 1. — eAppendix 1. Characteristics of intervention and control hospitals eAppendix 2. Billing Code 99497 eAppendix 3. Tracheostomy Procedure Codes eAppendix 4. Mortality Risk Score [file jamahealthforum-e243172-s001.pdf]

# Supplemental Online Content

Sacks OA, Murphy M, O'Malley J, Birkmeyer N, Barnato AE. A Quality Improvement Initiative for Inpatient Advance Care Planning. *JAMA Health Forum.* 2024;5(10):e243172. doi:10.1001/jamahealthforum.2024.3172

**eAppendix 1.** Characteristics of intervention and control hospitals

**eAppendix 2.** Billing Code 99497

**eAppendix 3.** Tracheostomy Procedure Codes

**eAppendix 4.** Mortality Risk Score

This supplemental material has been provided by the authors to give readers additional information about their work.

© 2024 Sacks OA et al. *JAMA Health Forum*.

**eAppendix 1.** Characteristics of intervention and control hospitals

| | **Control Hospitals (N=1,691)** | | **Hospitals staffed by intervention practitioners (N=109)** | | |
|---|---|---|---|---|---|
| | N | % | N | % | p-value |
| **Teaching Status** | | | | | |
| Non-teaching | 611 | 36.1% | 39 | 35.8% | |
| Teaching | 1,080 | 63.9% | 70 | 64.2% | 0.9408 |
| **Locality** | | | | | |
| Urban | 1,394 | 82.4% | 91 | 83.5% | |
| Rural | 297 | 17.6% | 18 | 16.5% | 0.7798 |
| **Hospital type** | | | | | |
| Non-federal, government | 192 | 11.4% | 10 | 9.2% | |
| Non-government, not for profit | 1,230 | 72.7% | 84 | 77.1% | |
| For profit | 269 | 15.9% | 15 | 13.8% | 0.6080 |
| **Bed size** | | | | | |
| <100 | 320 | 18.9% | 14 | 12.8% | |
| 100-499 | 1,135 | 67.1% | 82 | 75.2% | |
| 500+ | 236 | 14.0% | 13 | 11.9% | 0.1882 |
| **Additional services** | | | | | |
| Inpatient palliative care unit | 260 | 15.4% | 20 | 18.3% | 0.4065 |
| Palliative care program | 1,065 | 63.0% | 75 | 68.8% | 0.2211 |
| Hospice | 497 | 29.4% | 39 | 35.8% | 0.1574 |

© 2024 Sacks OA et al. *JAMA Health Forum*.

**eAppendix 2.** Billing Code 99497

Advance care planning including the explanation and discussion of advance directives such as standard forms [with completion of such forms, when performed], by the physician or other qualified health care professional; first 30 minutes, face-to-face with the patient, family member(s), and/or surrogate) or 99498 (each additional 30 minutes). Conversations are required to be at least 16 minutes in length to meet billing requirements. These conversations can be completed as many times as necessary by physicians and non-physician practitioners (e.g., advance practice providers). There are no place-of-service limitations, and these services are not limited to a particular physician specialty.  Any evidence of billing during the hospitalization was categorized as a binary variable during the stay, although ACP could be billed multiple times throughout a stay

© 2024 Sacks OA et al. *JAMA Health Forum*.

**eAppendix 3.** Tracheostomy Procedure Codes

Tracheostomy was identified using procedure codes 0B110F4, 0B110Z4, B113F4, B113Z4, B114F4, B114Z4 on the Medpar record. Intensive care utilization was identified using indicators of intensive and coronary care on the Medpar record. Gastrostomy was identified using procedure codes 0DH60U, 0DH60UZ, 0DH63U, 0DH63UZ, 0DH64U, 0DH64UZ, 0DH67U, 0DH67UZ, 0DH68U, 0DH68UZ, 0DH50UZ, 0DH53UZ, 0DH54UZ, 0DH8OUZ, 0DH83UZ, 0DH90UZ, 0DH93UZ, 0DH95UZ, 0DHA0UZ, 0DHA3UZ, 0DHA4UZ, 0DHA3UZ, 0DHA4UZ, 0DHB0UZ, 0DHB3UZ, 0DHB4UZ, 0DH57UZ, 0DH58UZ, 0DH84UZ, 0DH87UZ, 0DH88UZ, 0DH97UZ, 0DH98UZ, 0DHA7UZ, 0DHA8UZ, 0DHB7UZ, 0DHB8UZ on the Medpar record. We identified non-invasive mechanical ventilation using procedure codes 5A09, 5A09357, 5A09457, 5A09557 and invasive mechanical ventilation using procedure codes 5A19, 5A1935Z, 5A1945Z, 5A1955Z on the Medpar record. We used the destination code on the Medpar record to identify discharge to hospice (codes 50 and 51) and inpatient death (codes 20, 40, 41 and 42.) Days to death post-admission was calculated using the difference between the admission date and the date of death, ascertained from the Master Beneficiary Summary File (MBSF) with the difference being the censoring time if the patient had yet to die at the end of the follow-up; binary indicators for 30-day and 365-day post-admission death were created from this day count. We identified DNR orders using diagnosis code Z66 on the Medpar record and determined whether the order was present on admission using the present on admission indicator code Y; all DNR orders without a present on admission indicator were classified as newly initiated orders.

© 2024 Sacks OA et al. *JAMA Health Forum*.

**eAppendix 4.** Mortality Risk Score

The predicted mortality risk score incorporated age at time of admission and patient sex, admitting diagnosis, admission status, ICU utilization, comorbidities, emergency department visits within the last year, home oxygen use in the last year, 30-day urgent readmission status, transfers to and from the hospital, or whether patients were dependent on nursing care in the 30 days prior to admission (skilled nursing facility, hospice, home nursing care) (See appendix for details on these variables). We obtained a predicted probability mortality risk score associated with each stay by using a logistic regression that used the predictors previously described to model 365-day post-admission death.

| Variable | Source | Details |
|---|---|---|
| Admitting diagnosis | CMS hierarchical condition categories (CMS-HCC) | |
| Admission status | MedPAR | • Elective: 3 – elective or missing<br>• Urgent: 1 (emergency), 2 (urgent), 5 (trauma) or if the patient was admitted through the emergency department (revenue center codes 0450-0459, 0981 on the inpatient or outpatient claim or through an emergency room charge amount greater than $0 on the Medpar record) |
| ICU utilization | MedPAR | Indicators of intensive and coronary care on the MedPAR record |
| Comorbidities | Charlson Comorbidity Index definitions from all available inpatient claims | Myocardial infarction, congestive heart failure, peripheral vascular disease, cerebrovascular disease, dementia, chronic respiratory disease, mild liver disease, diabetes with and without complications, hemi/paraplegia, renal disease, non-metastic cancer, moderate to severe liver disease, metastatic cancer and HIV/AIDS |

© 2024 Sacks OA et al. *JAMA Health Forum*.

| | | |
|---|---|---|
| History of emergency department visits within the last year | Inpatient or outpatient claims | Revenue center codes 0450-0459, 0981 or through an emergency room charge amount greater than $0 on the Medpar record |
| Oxygen use within the last year | Durable medical equipment claims | HCPCS codes E1390, E1391, E0424, E0431, E0433, E0434, E0439, E1405, E1406, E1392, E0441, E0442, E0443, E0444, K0738 on durable medical equipment claims |
| 30-day urgent readmission status | MedPAR | Stay with an admission date occurring within 30 days after the discharge date on a previous Medpar record in patients with an urgent admission |
| Home with services | MedPAR | • Point of origin code 4 for in-hospital transfer and 5 for out of hospital transfer |
| Dependent on services, residing in a nursing home, on hospice within 30 days leading up to admission | Skilled nursing facility, home health agency, hospice claims, nursing home care | nursing home care (identified using HCPCS codes 99304-99310, 99315, 99316, 99318 on Part B claims) |

© 2024 Sacks OA et al. *JAMA Health Forum*.
