# Supplementary material for: A Quality Improvement Initiative for Inpatient Advance Care Planning
Source: JAMA Health Forum. 2024 Oct 4;5(10):e243172. doi: 10.1001/jamahealthforum.2024.3172 (PMC11452818; doi:10.1001/jamahealthforum.2024.3172)
Supplement: Supplement 2. — Data Sharing Statement [file jamahealthforum-e243172-s002.pdf]

Data Sharing Statement

Sacks. A Quality Improvement Initiative for Inpatient Advance Care Planning. *JAMA Health Forum*. Published October 04, 2024. doi:10.1001/jamahealthforum.2024.3172

Data
Data available: No
